# Magnetic Resonance-guided High Intensity Focused Ultrasound in the presence of biopsy markers

**DOI:** 10.1186/s40349-017-0103-1

**Published:** 2017-09-20

**Authors:** Charles Mougenot, Chrit Moonen

**Affiliations:** University Medical Center Utrecht, Heidelberglaan 100, Room Q03.4.21, 3584 CX Utrecht, The Netherlands

**Keywords:** Markers, MRI, Ultrasound, HIFU, Thermometry

## Abstract

**Background:**

Magnetic Resonance guided High Intensity Focused ultrasound (MR-HIFU) offers precise non-invasive thermotherapy for clinical applications such as the treatment of breast lesions. However, patients with a biopsy marker are usually not eligible for MR-HIFU treatment. This study investigates the interaction of some MR-compatible markers with MR-HIFU thermotherapy.

**Methods:**

The MR-HIFU compatibility of 14 markers (6 Gold Anchor and 4 Visicoil markers in gold, 1 Visicoil marker in brass, 3 BiomarC markers in carbon coated) were tested using the Sonalleve breast MR-HIFU platform at 1.5 T. The impact of these markers was assessed by counting the number of voxels with low signal intensity on MR thermal maps and by comparing temperature increases induced by the HIFU beam.

**Results:**

Most markers were visible on thermal maps with an apparent size 4.2 ± 3.1 and 2 ± 1.8 times larger than their respective actual width and length. The volume of masked voxels was for most of the markers much larger than the actual volume of the marker (up to a factor 65.1). However, it represents only a small fraction of the 12 mm diameter targeted region (up to 8.8 voxels which represents 19% of this targeted region). Some differences in the maximal temperature increase were observed especially for BiomarC 1 × 3 and BiomarC 2 × 4 markers enhancing the heating. These differences were less pronounced at the edge of the targeted region.

**Conclusion:**

All markers had a minimal impact on the volume above the thermal dose threshold of 240 EM since the differences measured were smaller than the in-plane image resolution of 1.56 mm.

## Background

The detection of microcalcifications during screening mammography prompts biopsy of the abnormality and insertion of a marker at the biopsy location. Where biopsies are shown to be positive for tumour the marker has an essential role; 1) to prepare the placement of radioactive (iodine) markers under ultrasound guidance for subsequent surgery and 2) for follow-up to localize the region which has already been subject to biopsy and histopathological examination. As a consequence, radiographic markers have been developed to be easily visible on mammography and ultrasound images [1] but not necessarily on MR images. Breast MR images with standard-of-care titanium marker show a large artifact [2, 3] in conventional MRI (limiting its use in diagnosis), and in particular in the typical gradient-echo MRI sequences used for MR thermometry maps, due to their difference in magnetic susceptibility as compared to tissue. With the increased need for MRI based diagnostics and therapy planning, new MR-compatible biopsy markers made of gold, brass or carbon are now commercially available.

MR-HIFU uses high-intensity focused ultrasound (HIFU) guided by Magnetic Resonance (MR) to focus ultrasonic energy deep in the body without incisions or radiation. MR-HIFU treatment offers very precise spatial control with an accuracy of a few millimetres [4, 5]. A detailed description of the clinical applications of therapeutic ultrasound is available on https://www.fusfoundation.org/. MR guided therapeutic ultrasound is predominantly used for thermotherapy of the prostate cancer [6], uterine fibroid [4, 7], liver cancer [8], breast cancer [9, 10], bone metastasis [11] and osteoid osteoma [12]. However, MR-HIFU treatment of small breast tumors or Ductal Carcinoma In Situ (DCIS) lesions has not been considered primarily because the presence of a metallic implant is considered an exclusion criterion for MR-HIFU^9^. These markers are systematically placed during breast biopsy following detection of a suspicious lesion during screening mammography.

This paper describes an evaluation of the compatibility of some MR-compatible markers with therapeutic ultrasound. For this purpose, 14 different MR-compatible markers manufactured by three companies were tested in a phantom mimicking soft tissue. Typical clinically used sonication patterns were applied on these markers. The impact of these markers on the number of voxels with low signal intensity on the MR thermal maps and the resulting heating were analyzed relatively to a reference phantom without marker.

## Methods

### Experimental setup

**Fig. 1:**
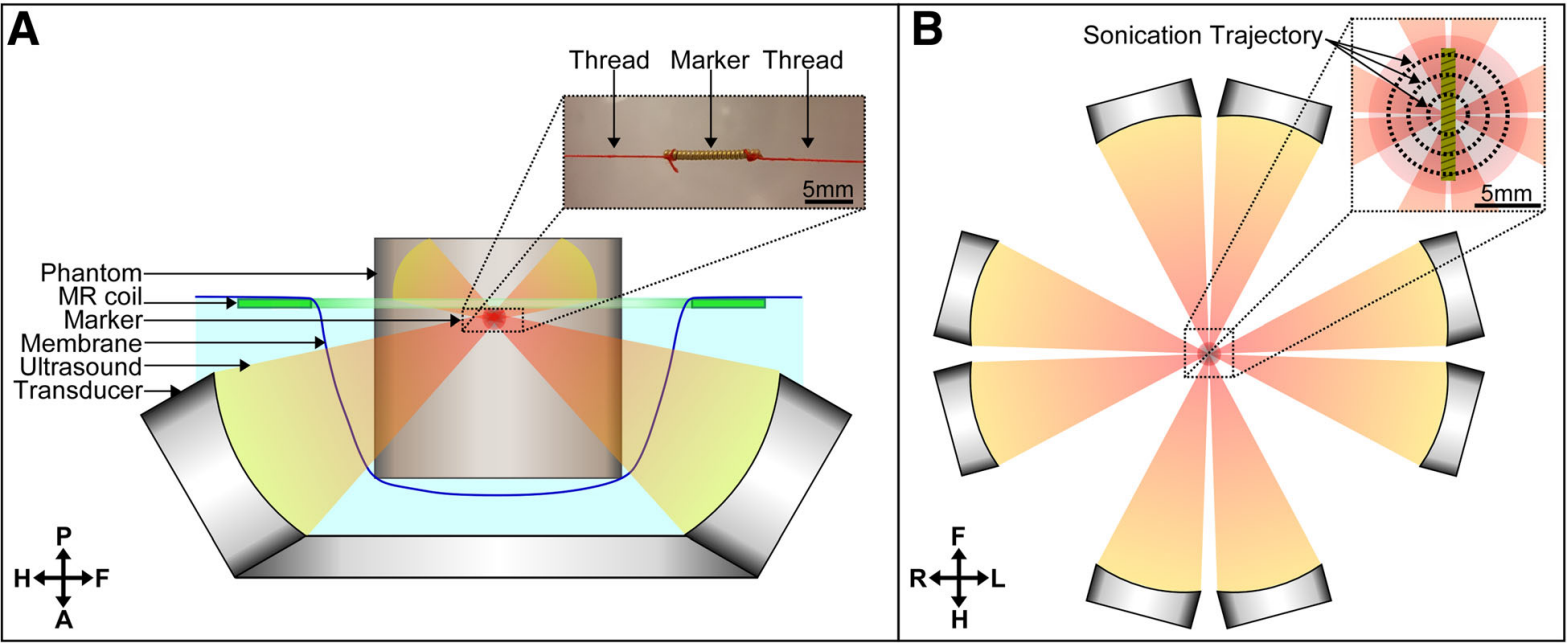
Side view (**a**) and top view (**b**) of the experimental setup with the Sonalleve breast MR-HIFU platform emitting ultrasound waves converging inside a phantom with an embedded marker. The sonications trajectory composed of 3 horizontal concentric circles was centered on the marker

The experimental setup is presented in Fig. 1a; Experiments were conducted with a prototype Sonalleve breast MR-HIFU platform (Profound, Mississauga, Canada) which is designed to emit ultrasound into the breast horizontally to avoid the risk to damage vital organs while treating breast lesions [13, 14]. This system is composed of a three-axis motorized system to align a transducer with the targeted region. The transducer is a phased array composed of 256 piezo-composite elements of 6.6 mm diameter operating at 1.45 MHz (Imasonic, Voray sur l’Ognon, France). These elements are distributed within 8 modules composed of 32 elements (Fig. 1b) placed along a circle surrounding the breast cup made of 70 μm PETG membrane which is acoustically transparent. The ultrasound waves converge at the center of this breast cup to form a focal point at −6 dB of 0.7 × 0.7 × 4.5 mm^3^, and to induce a temperature increase which was monitored by a 1.5 T Achieva MRI (Philips, Best, Netherlands). Each tested marker was embedded into a cylinder gel of 8 cm diameter and 8 cm height composed of 3% agar and 2% silica to mimic acoustic properties of soft tissues [15]. These markers were centered and oriented within this phantom using red wiring threads of 250 μm attached at each extremity of the marker as shown in Fig. 1a. This phantom was placed inside the breast cup and acoustically coupled to the transducer with degassed water.

Table 1 describes the 14 MR-compatible markers tested; 6 Gold Anchor markers (Naslund Medical AB, Huddinge, Sweden) made of an alloy of gold and 0.5% pure iron; 5 Visicoil markers (IBA Dosimetry, Schwarzenbruck, Germany) made of gold or brass; and 4 BiomarC markers (Carbon Medical Technologies, Minnesota, USA) made of pyrolytic carbon coated zirconium oxide. Because the Gold Anchor markers have a flexible geometry they were tested in two configurations; linear shape or ball shape. In this study, each marker was identified by their brand name and their size in mm (width and length). The volume of each marker was estimated using a cylinder model (or spherical model for ball shape Gold Anchor marker). These markers had a width ranging from 0.28 mm to 2 mm, a length ranging from 3 mm to 30 mm and a volume ranging from 0.62 ml to 12.57 ml. The markers Gold Anchor 0.28 × 10, 0.28 × 20 or 0.4 × 20 with a linear shape are the same markers as the markers Gold Anchor 1.3 × 1.3, 1.8 × 1.8 or 2.1 × 2.1 reconfigured in a ball shape.

**Table 1:**
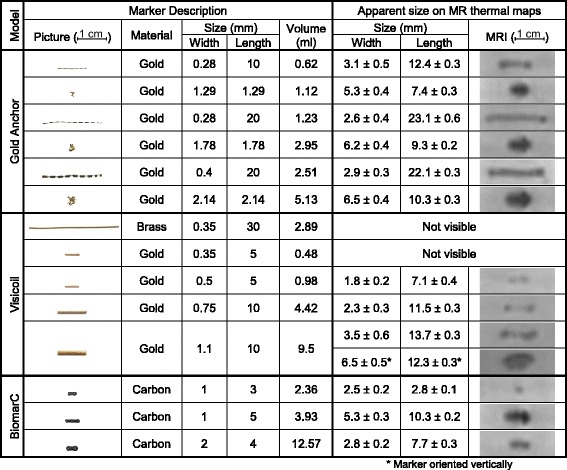
Description of MR-compatible markers used and their apparent size on MR thermal maps

^*^Marker oriented vertically

As reported in the literature [16–18], the orientation of elongated devices (i.e catheters and needles) plays also an important role in the size of the artifact; The largest artifacts (or signal voids) were systematically observed with an orientation orthogonal to the magnetic field B_0_. For this reason, all the markers were tested aligned with the magnetic field (i.e foot-head direction) to improve the analysis of the temperature at proximity of these markers. However, the largest elongated marker Visicoil 1.1 × 10 was also tested in vertical orientation (i.e anterior-posterior direction) to assess the worst case.

A total of 17 phantoms were made; 15 phantoms with a marker (the 14 markers listed in Table 1 with one marker tested in two different orientations) and 2 reference phantoms without markers. The first reference phantom was only composed of agar-silica gel. The second reference was also composed of agar-silica gel and included a wire thread.

### Methodology

MR thermal maps were acquired with an EPI echo gradient sequence (TE = 20 ms, TR = 85 ms, Flip angle = 20, EPI factor = 19) with a field of view of 200 × 200 mm^2^ and a voxel size of 1.56 × 1.56 × 5 mm^3^.

In a first part the size (width and length) of the marker was measured manually using a coronal slice (or sagittal for the Visicoil 1.1 × 10 marker in vertical position) averaged by a factor 32 to reduce the noise level. These measures were repeated three times for each marker with the phantom removed and repositioned between each measurement.

In a second part HIFU heating was performed on each phantom. The sonication pattern was based on a displacement of the focal point in a coronal plane along inner-outer circles of 3, 6 and 9 mm with an acoustic power of 40 Wac applied during 30s. These horizontal concentric circles were centered on the marker as illustrated in Fig. 1b. The sonication was initiated 10 s after the start of the acquisition of MR thermal maps to allow the quantification of the number of voxels masked by markers prior to temperature increase. These sonication parameters were selected to reproduce typical heating pattern used during clinical applications [19]: a maximal temperature increase of 30 °C with an ablated volume of 0.6 ml (ellipsoidal volume of 12 × 12 × 8 mm^3^). This sonication pattern is slightly larger than the one used during the first clinical trial performed with this Sonalleve breast MR-HIFU platform (i.e inner-outer circles of 3 and 6 mm only) but this clinical trial was designed to ablate only a small region at the center of the tumor rather than the complete tumor. This sonication pattern is in the lower size range of the possible volumetric ablations which can reach up to several ml^4^. Each sonication was repeated 12 times in each phantom with 6 sonications centered on the markers and 6 sonications located 15 mm below the markers. Only 4 sonications were performed on the Visicoil 1.1 × 10 marker in the horizontal position due to the limited availability of this marker. The sonications located below the marker were used to compare the absorption coefficient between the different gels and to define a compensation factor for this variability of the absorption coefficient. After each sonication, the phantom was removed and replaced by another phantom. All sonications were performed in a random order with a least 1 hour between two successive sonications within the same phantom to ensure complete cooling of the phantom before the start of another heating.

For each heating, the temperature was monitored in a coronal plane and a sagittal plane intersecting the targeted location. The temperature was monitored during the sonication duration and 1 minute after because the cooling period also contributed to the thermal dose buildup. The impact of the marker on thermal map quality was quantified by a count of the number of voxels masked by the marker in the coronal slice. Voxels were considered masked when the intensity of the magnitude signal was below a threshold equivalent to a temperature standard deviation of 2 °C. The relation between the intensity of the magnitude signal and the temperature standard deviation was based on the quantification of the signal to noise ratio [20]. Because the number of masked voxels slightly changed for each image time frame, only the voxels which were masked for more than half of the image time frames were counted. This count of the total number of voxels masked was repeated for each of the 6 sonications performed at the marker location.

MR thermal maps were processed using the Proton Resonance Frequency (PRF) relation [21, 22] to convert the phase variation in a temperature variation. To improve the accuracy of the temperature mapping, image time frame were averaged by a factor 4 over time. For the sonications acquired away from the markers, a PRF coefficient of 0.0094 ppm/°C was used. The average temperature change observed over a region of 3 × 3 voxels was used to obtain a relative quantification of the ultrasound absorption coefficient of each phantom. This averaged temperature increase was also averaged over 6 sonications and divided by the average temperature increase found within all phantoms. This method provided a correction coefficient of the difference of the ultrasound absorption coefficient between phantoms and was found to be equal to 100 ± 4.6% [92.1%; 106.7%]. For the sonications located on a marker (or equivalent location for reference phantoms), the PRF coefficient of 0.0094 ppm/°C divided by this correction coefficient was used. This correction was based on the assumption that the temperature increase is proportional to the absorption coefficient as defined by the bioheat transfer equation [23].

The impact of the marker on the heating pattern was evaluated by two criteria; the maximal temperature increase and the thermal dose volume above 240 EM. These two metrics were used for each of the 6 sonications performed on each marker.

The maximal temperature was selected to be the largest temperature measured in the coronal plane within the voxels not masked (i.e. with a temperature standard deviation lower than 2 °C without averaging or lower than 1 °C with a temporal averaging by a factor of 4). The temperature increase at the edge of the targeted region was also analyzed by averaging the temperature in the 28 voxels located between circle of 9 mm diameter (i.e the outer sonication trajectory) and 12 mm diameter (i.e the typical diameter reaching a thermal dose above 240EM). The maximal temperature increase and the temperature increase at the edge were compared for each marker to the temperature measured in the reference phantom using a t-test.

Before processing the thermal dose, the voxels masked in the thermal maps were replaced by the average of value of neighboring voxels. The sagittal and coronal thermal maps were used to reconstruct a distribution of the temperature in three dimensions using a compressed sensing algorithm. These reconstructed thermal maps were used to process the three dimensional distribution of the thermal dose [24]. Despite the fact that the phantom was initially at the room temperature of 20 °C, a baseline temperature of 37 °C was used to process the thermal dose in similar conditions as the ones encountered for patients. The thermal dose volume above 240 EM was obtained by a count of the voxels above this threshold.

An additional experiment was conducted to validate the MR thermometry at vicinity of the Visicoil 0.75 × 10 marker using three thermocouples placed in the middle, at the border and 5 mm away from this marker. T-type thermocouples with a tip of 0.25 mm diameter and 1 mm length were used to minimize the signal void induced by thermocouples. Sonication centered on the thermocouples was not possible due to ‘viscous heating’ at the thermocouple–tissue interface. This heating is arising from the difference in density between the thermocouple wire and the surrounding tissue which leads to relative motion between the two [25]. As suggested in the literature to reduce the influence of the ‘viscous heating’, the center of the sonication was placed 1 cm away from the center of the marker and the first 5 s sonication was removed from the analysis. To explore a large of temperature variation at the thermocouple locations, the acoustic power level was increased to 120 Wac and the sonication duration was increased to 60 s.

## Results

### Apparent markers size

Table 1 displays an example of an MR image for each marker with the MR thermal map sequence with temporal averaging by a factor 32 to better distinguish the marker. The markers appear blurry on these images due to the low spatial resolution (1.56 × 1.56 × 5 mm^3^) compared the width of marker which is submillimetric for most of them. Despite the averaging factor 32, the markers Visicoil 0.35 × 30 and Visicoil 0.35 × 5 were not visible on MR thermal maps because these markers affected at most 1.2% of the voxel volume. However, the Visicoil 0.35 × 5 marker which is frequently used at UMC Utrecht for breast cancer biopsy, is visible on other clinical MR sequences with a smaller isotropic resolution of 1 mm. All other markers were visible on MR thermal maps, including the Gold Anchor 0.28 × 10 marker which has a smaller diameter than the Visicoil 0.35 × 5 marker. The apparent width and length of the markers on MR image were on average 4.2 ± 3.1 and 2 ± 1.8 times larger than their respective actual width and length. As previously established [16–18], for elongated markers their apparent size depends on the orientation of the marker relatively to the main magnetic field B_0_. As a consequence, the Visicoil 1.1 × 10 marker has a much larger apparent width of 6.5 mm when orientated vertically (i.e orthogonal to B_0_) compared to the width of 3.5 mm when orientated horizontally (i.e parallel to B_0_).

### Number of masked voxels

**Fig. 2:**
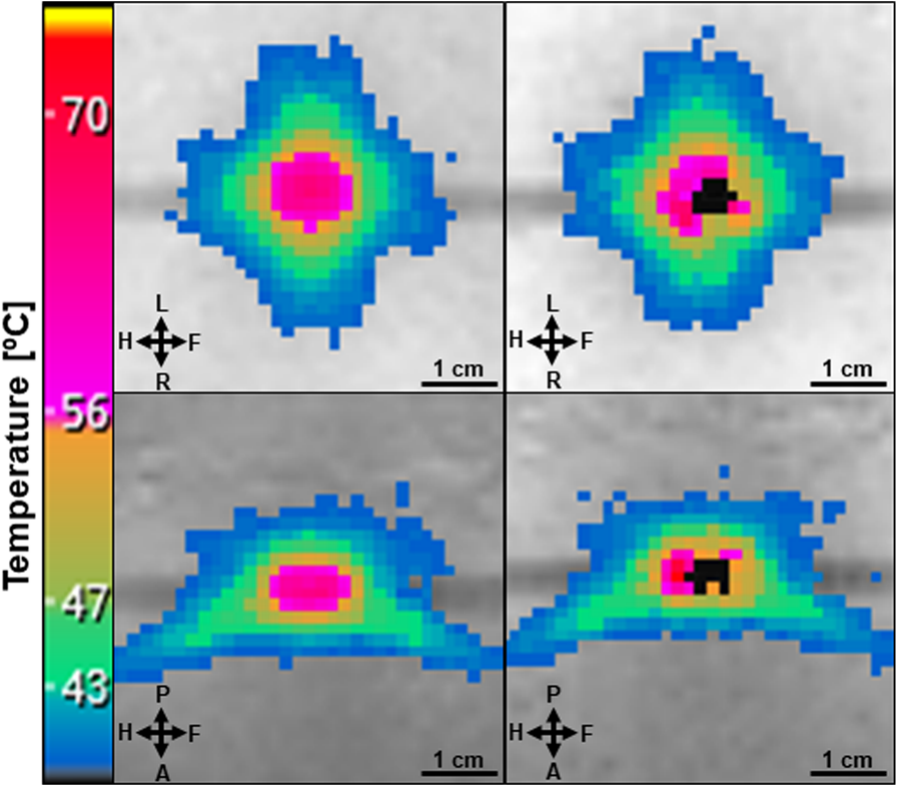
Thermal maps at the end of a sonication in coronal (up) and sagittal (bottom) slices inside the reference phantom (left) and the phantom with Gold Anchor 2.1 × 2.1 marker (right)

Figure 2 presents thermal maps acquired at the end of a sonication performed in the reference phantom and in the phantom with Gold Anchor 2.1 × 2.1. In both cases the temperature distribution appeared similar, except that the temperature information is missing in a few voxels at the marker location. The Gold Anchor 2.1 × 2.1 marker was selected to illustrate the impact of the marker on thermal maps because it is the marker which induced the largest number of voxels masked on thermal maps.

**Fig. 3:**
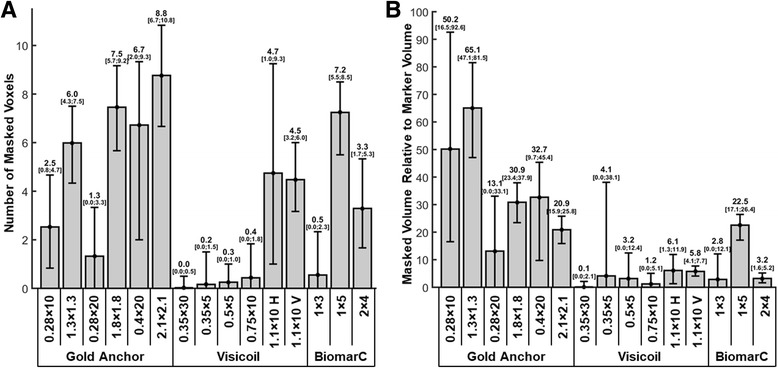
Number of masked voxels (**a**) and masked volume relative to marker volume (**b**) on the coronal thermal maps for each marker using a threshold equivalent to a temperature standard deviation of 2 °C

For a more quantitative comparison, Fig. 3a displays the average number of masked voxels on the coronal thermal maps for each marker. Figure 3a provides in brackets, the typical minimum and maximum number of masked voxels occurring during one experiment. Most of the smallest markers (Visicoil 0.35 × 30, Visicoil 0.35 × 5, Visicoil 0.5 × 5, Visicoil 0.75 × 10 and BiomarC 1 × 3) were masking in average less than one voxel. Most of the largest markers (Gold Anchor 1.8 × 1.8, Gold Anchor 0.4 × 20, Gold Anchor 2.1 × 2.1 and BiomarC 1 × 5) were masking the largest number of voxels. The Gold Anchor markers in ball configurations masked more voxels than the same markers in linear configuration. The Visicoil 1.1 × 10 marker with a vertical orientation (identified with the letter ‘V’ on Fig. 3) masked a similar number of voxels than the same marker with an horizontal orientation (identified with the letter ‘H’ on Fig. 3). However, the same analysis conducted in the sagittal slice indicates that this marker masked less voxels with the horizontal orientation (4.4 voxels) than the vertical orientation (9.6 voxels).

Figure 3b presents the ratio of the volume of masked voxels by the volume of the marker (listed in Table 1). The largest masked volume ratio was 35.5 ± 19.2 for Cold Anchor markers, followed by BiomarC markers with a ratio equal to 9.5 ± 11.2. The smallest ratio of 3.4 ± 2.4 was obtained with Visicoil markers.

The volume of masked voxels was for most of the marker much larger than the actual volume of the marker and reached up to a factor 50.1 and 65.1 for the smallest Gold Anchor markers. However, the number of masked voxels remained small compared to the total number of voxel located within the targeted region (up to 8.8 voxels out of 46 voxels or 19% of the surface).

**Fig. 4:**
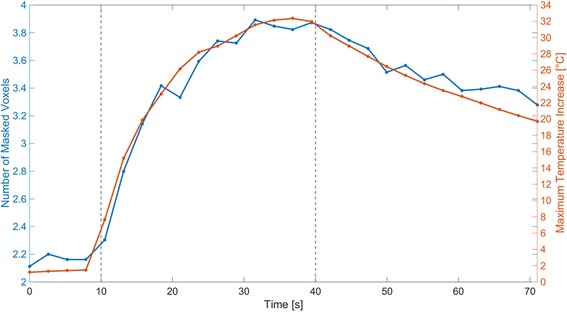
Number of masked voxels (blue curve) and the maximal temperature increase (red curve) averaged over all markers. Grey doted lines indicate the start and the end of the sonication at 10 s and 40 s

To further analyze the variability of the number of masked voxel over the course of an experiment (minimum and maximum values reported in brackets Fig. 3), the Fig. 4 displays as a blue curve the number of masked voxels for each time frame using the average over all markers. As comparative purpose, on the same figure the maximum temperature increase for each time frame is displayed as red curve using also the average over all markers. The number of masked voxels is proportional to the maximum temperature increase in the targeted region with a correlation R [2] equal to 0.98. As consequence, the number of masked voxel increased by a factor 1.8 between the start and the end of a sonication.

### Impact on the heating pattern

**Fig. 5:**
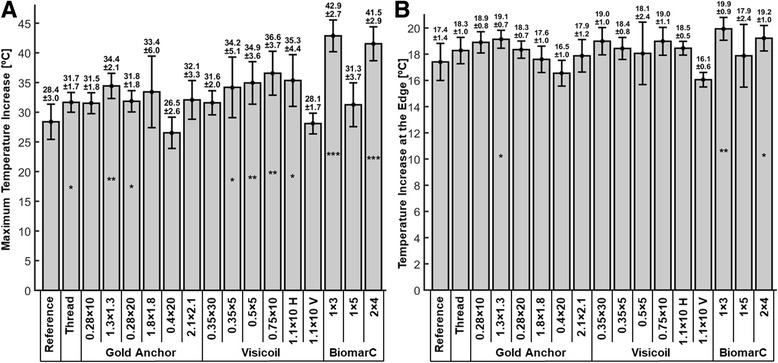
Maximum temperature increase (**a**) and average temperature increase at the edge of the targeted region (**b**) for each marker. Symbols *, ** and *** correspond to a *P*-value inferior to 0.05, 0.01 and 0.001

Figure 5a presents the maximal temperature increase measured upon sonication of the phantom for each marker. In the reference phantom, the temperature increase was 28.4 ± 3 °C. The presence of the wire thread augmented slightly the maximum temperature increase up to 31.7 ± 3 °C. The maximal temperature increase for each marker ranged from 26.5 °C to 42.9 °C. The most significant differences relative to the phantom reference were observed for BiomarC 1 × 3 and BiomarC 2 × 4 markers with the largest temperature increase of 42.9 °C and 41.5 °C which corresponds to 51% and 46% more than the reference value.

Figure 5b shows the temperature increase measured at the edge of the targeted region for each marker. Compared to the maximal temperature increase, less variability was observed for temperature increase at the edge ranging from 16.9 °C to 19.9 °C. Largest temperature increases at the edge were also observed for BiomarC 1 × 3 and BiomarC 2 × 4 markers with a value of 14% and 10% above the reference value.

**Fig. 6:**
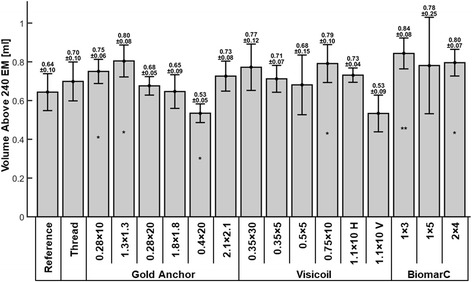
Volume above 240 EM in ml for each marker. Symbols *, ** and *** correspond to a P-value inferior to 0.05, 0.01 and 0.001

Figure 6 presents the volume reaching a thermal dose above 240 EM. The volume above 240 EM in the reference phantom is 0.64 ± 0.1 ml. The difference of volume above 240 EM for each marker is in the same range as the experimental reproducibility. As a consequence, few conclusions can be made about the fact that one marker might impact more than another marker the volume above 240 EM. The volume above 240 EM for the phantoms with markers range from 0.53 ml to 0.84 ml which represents a volume variation between −17% and 31% relative to the reference. This volume variation can also be translated as a variation of the ablated diameter between −6% and 9.4%. For a typical diameter of the ablated region of 12 mm in the coronal plane, this ablated diameter variation is equal to a change between −0.7 mm and 1.1 mm which is less than the in-plane resolution of 1.56 mm. Despite the uncertainty related to the image resolution, the orientation of the marker Visicoil 1.1 × 10 seems to have an impact on the heating: relative to the horizontal orientation, the vertical orientation of this marker reduces the maximal temperature increase by 20% and the volume above 240 EM by 27%.

**Fig. 7:**
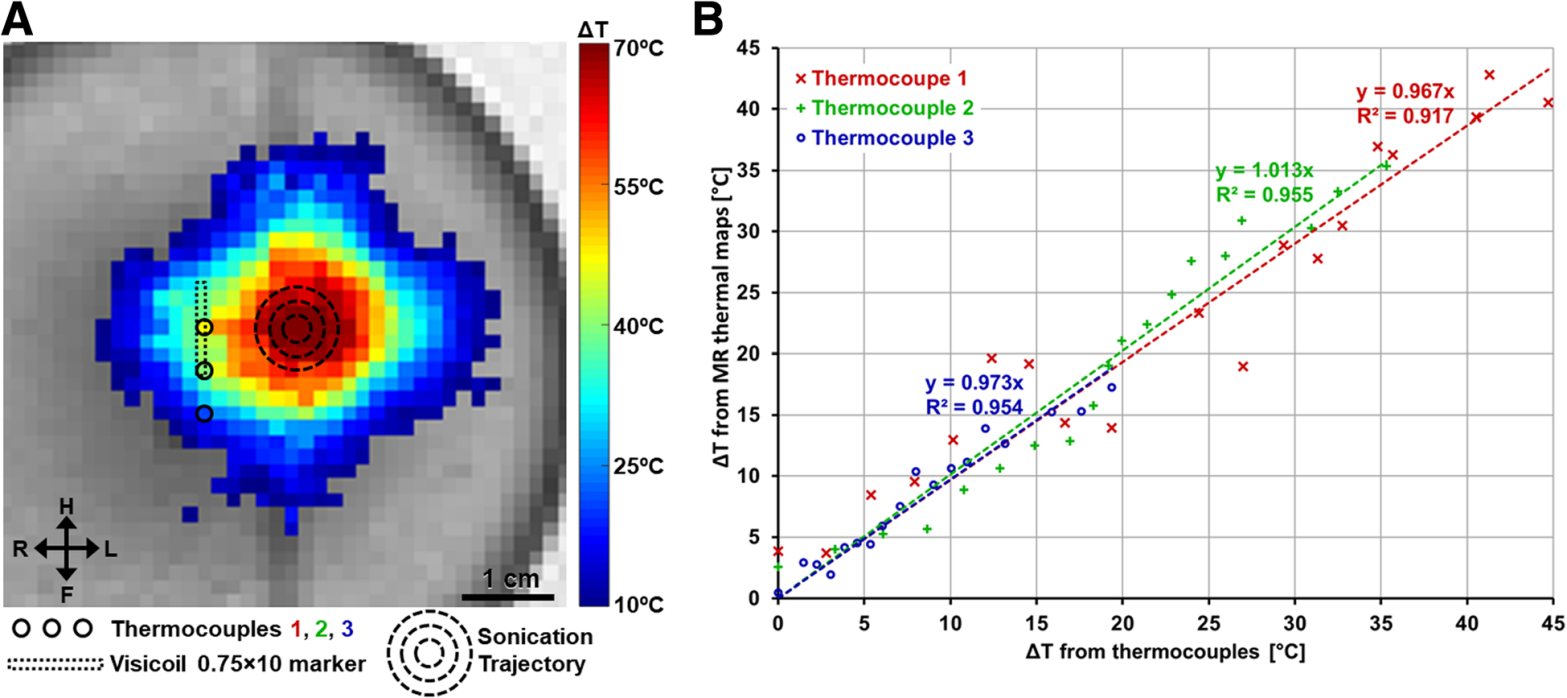
(**a**) Thermal map acquired at the end of a sonication 60 s and the relative position of the marker and three thermocouples. (**b**) Correlation between temperature variation measured from the MR thermal maps and the three thermocouples

To validate the MR thermometry at proximity of a marker, Fig. 7a shows the location of the Visicoil 0.75 × 10 marker and the three thermocouples relatively to the sonication trajectory on top of the MR thermal map acquired at the end of the sonication. Figure 7b indicates that the temperature variation from MR thermal maps corresponds to the temperature measured by thermocouples with a slope between 0.973 and 1.013 and a correlation R^2^ between 0.92 and 0.96.

## Discussion

The lack of visibility of the markers Visicoil 0.35 × 30 and Visicoil 0.35 × 5 on MR thermal maps can be considered as an advantage since no voxel was masked. The fact that the Gold Anchor 0.28 × 10 marker, with a smaller diameter than the Visicoil 0.35 × 5 marker, is visible on thermal maps might be due to the fact the Gold Anchor are based on an alloy with 0.5% iron [26, 27]. For the same reasons, the masked volume relative to the marker volume was much larger for Gold Anchor markers than the Visicoil markers.

The fact that the number of masked voxels is correlated with the temperature increase in the target region was mainly due to the T1 change. When the temperature rises, the T1 increases by 1%/C which causes a decay of the MR signal intensity and an SNR drop [28].

It was expected that vertical orientation of the Visicoil 1.1 × 10 marker induce a larger signal void [26] than the horizontal orientation. This effect was confirmed in the sagittal slice but not in the coronal slice. This might be explained by the fact that Visicoil 1.1 × 10 marker was twice longer than the slice thickness and thus half of this marker was located outside of the coronal slice when orientated vertically.

The impact of the marker on the heating didn’t appear to be related to the size of the marker; The large markers Gold Anchor 0.4 × 20, Visicoil 1.1 × 10 and BiomarC 1 × 5 were associated with the smallest maximal temperature increase (Fig. 5). However, a large number of voxels were masked for these large markers and it is likely that a larger temperature increase occurred at the location of these masked voxels.

From a clinical point of view, the volume above 240 EM is more important than the maximal temperature increase as it is the most frequently used parameter to predict the therapeutic outcome with the non-perfused volume [29, 30]. Even if it has been established that thin metal wires such as thermocouples induce viscous heating at its proximity [25], the markers had a minimal impact on the size of the ablated volume because voxels located at the border of the ablated volume were sufficiently remote from the marker. For this study, a rather small but clinically relevant targeted volume was selected. Most probably, larger targeted volumes are less influenced by the marker because border voxels would be even more remote from the marker. Smaller sonication on a marker such as the initial test sonication (used to check the beam location) might be more problematic. In such case, it would probably be easier to perform test sonication at least 3 mm away from the marker (i.e outside of the apparent location of the marker on MR images).

It is also possible that the patient has a marker which is not centered in the targeted volume or that multiple markers are inserted in different regions of the breast due to multiple biopsies. The fact that the 6 markers (Gold Anchor 0.28 × 10, Gold Anchor 0.28 × 20, Visicoil 0.35 × 30, Visicoil 0.75 × 10 and Visicoil 1.1 × 10) longer than the outer sonication trajectory (as shown in Fig. 1b) have not induced a significant difference of the temperature increase at the edge (as shown in Fig. 5b) suggests that markers located at the edge of the trajectory have minor influence on the heating. Similarly, the symmetry of the temperature distribution shown Fig. 7a, indicates that the Visicoil 0.75 × 10 marker placed 10 mm from the center of sonication trajectory (i.e 5.5 mm away from the outer circle) had a minimal impact on the heating pattern. Reference sonications performed 15 mm below each marker (i.e. outside the ultrasound beam path) did not indicate any temperature increase at the location of the marker.

The voxel size used for MR thermal mapping (1.5 × 1.5 × 5 mm^3^ for breast treatments and 2.5× 2.5 × 7 mm^3^ for pelvic treatments) is usually much larger than marker size. Due to a partial volume effect within one voxel, a larger temperature increase than the one monitored by the MRI might occur at the surface of the marker due viscount heating [25]. Overheating at the surface of marker located in the tumor might not be problematic for thermal ablation, but it can be problematic for hyperthermia applications requiring a precise temperature control of 1 °C. This study was conducted using a magnetic field of 1.5 T, further investigation should be conducted using a higher field (3 T or 7 T) since the apparent size of markers and the number of masked voxels tend to increase with the magnetic field strength.

## Conclusion

In this study, the MR-HIFU compatibility of 14 markers (6 Gold Anchor and 4 Visicoil markers in gold, 1 Visicoil marker in brass, 3 BiomarC markers in carbon coated) with a width ranging from 0.28 mm to 2 mm, a length ranging from 3 to 30 mm and a volume ranging from 0.62 ml to 12.57 ml were assessed. Most of these markers were visible on thermal maps with an apparent size 4.2 ± 3.1 and 2 ± 1.8 times larger than their respective actual width and length. The volume of masked voxels was for most of the markers much larger than the actual volume of the marker (up to a factor 65.1), however it represents only a small fraction of the 12 mm diameter targeted region (up to 8.8 voxels which represents 19% of this targeted region). Some differences in the maximal temperature increase were observed especially for BiomarC 1 × 3 and BiomarC 2 × 4 markers enhancing the heating. These differences were less pronounced at the edge of the targeted region. All markers had minimal impact on the volume above the thermal dose threshold of 240 EM since the differences measured were smaller than the in-plane image resolution of 1.56 mm.

## Abbreviations

DCIS: Ductal Carcinoma In Situ
HIFU: High intensity focused ultrasound
MR-HIFU: Magnetic resonance-guided high intensity focused ultrasound
MRI: Magnetic resonance imaging
